# Zhi-Zi-Chi Decoction Reverses Depressive Behaviors in CUMS Rats by Reducing Oxidative Stress Injury *Via* Regulating GSH/GSSG Pathway

**DOI:** 10.3389/fphar.2022.887890

**Published:** 2022-04-07

**Authors:** Yin Zhang, Yi-Chao Fang, Li-Xun Cui, Yue-Tong Jiang, Yu-Sha Luo, Wen Zhang, De-Xun Yu, Jun Wen, Ting-Ting Zhou

**Affiliations:** ^1^ School of Pharmacy, Second Military Medical University, Shanghai, China; ^2^ Shanghai Key Laboratory for Pharmaceutical Metabolite Research, School of Pharmacy, Second Military Medical University, Shanghai, China; ^3^ Chengdu Institute for Drug Control, Chengdu, China

**Keywords:** anti-depression, metabolomics, chronic unpredictable mild stress (CUMS), fructus gardeniae, GSH/GSSG pathway, zhi-zi-chi decoction (ZZCD)

## Abstract

Depression is one of the main diseases that lead to disability and loss of ability to work. As a traditional Chinese medicine, Zhi-zi-chi decoction is utilized to regulate and improve depression. However, the research on the antidepressant mechanism and efficacy material basis of Zhi-zi-chi decoction has not been reported yet. Our previous research has found that Zhi-Zi-chi decoction can reduce glutamate-induced oxidative stress damage to PC 12 cells, which can exert a neuroprotective effect, and the antidepressant effect of Zhi-Zi-chi decoction was verified in CUMS rat models. In this study, the animal model of depression was established by chronic unpredictable mild stimulation combined with feeding alone. The brain metabolic profile of depressed rats was analyzed by the method of metabolomics based on ultra-performance liquid chromatography-quadrupole/time-of-flight mass. 26 differential metabolites and six metabolic pathways related to the antidepressant of Zhi-zi-chi decoction were screened and analyzed. The targeted metabolism of the glutathione metabolic pathway was analyzed. At the same time, the levels of reactive oxygen species, superoxide dismutase, glutathione reductase, glutathione peroxidase in the brain of depressed rats were measured. Combined with our previous study, the antioxidant effect of the glutathione pathway in the antidepressant effect of Zhi-zi-chi decoction was verified from the cellular and animal levels respectively. These results indicated that Zhi-zi-chi decoction exerted a potential antidepressive effect associated with reversing the imbalance of glutathione and oxidative stress in the brain of depressed rats.

## Introduction

Depression is a common illness that severely increases the burden of disease worldwide and diminishes the quality of life. With a lifetime risk of over 15%, almost one in five people suffers one episode in their lifetime. Various factors contribute to the pathophysiology of depression, including the monoamine hypothesis, hypothalamic-pituitary-adrenal axis changes, neuroplasticity and neurogenesis, and inflammation (Malhi and Mann, 2018). Although pharmacotherapy for depression has changed into antidepressants with more selective actions, several defects such as the treatment-resistant phenomenon, the delay of drug efficacy, and serious side effects still affect patient outcomes. Hence, the efficacy and acceptability must be taken into consideration for new antidepressant discovery. In particular, traditional Chinese medicine has emerged as a potential area of exploration.

Fructus Gardeniae is the fruit of Rubiaceae plant Gardenia jasminoides J. Ellis, which belongs to the first batch of dual-use resources of medicine and food issued by the Ministry of health. It has the functions of protecting liver, benefiting gallbladder, reducing blood pressure, sedation, hemostasis and detumescence (Luo et al., 2021). Semen sojae praeparatum is a fermented product of mature seeds of Glycine max (L.) Merr, which is widely consumed as a kind of cooking and condiment (Guo et al., 2018). Zhi-zi-chi decoction (ZZCD), a traditional Chinese medicine (TCM) formula chronicled in Shang Han Lun, contains Fructus Gardeniae and Semen sojae praeparatum. ZZCD has been extensively used for the therapy of depression, febrile diseases, and agrypnia for over 1,000 years in China. According to current researches, iridoid glycosides and isoflavones are reported to be the main active ingredients in ZZCD, which show various pharmacological activities including anti-inflammation, antipyretic effect, and neuroprotection. Moreover, geniposide, one of the iridoid glycosides of ZZCD, reversed the chronic unpredictable mild stress (CUMS)-induce depressive behaviors in rats (Cai et al., 2015). Daidzein, one of the isoflavones of ZZCD, exerted antidepressant effect *via* alleviating the hypothalamic-pituitary-adrenal (HPA) axis dysfunctions (Wang et al., 2019), which suggested a strong link between ZZCD and antidepressant effect. Previous studies have shown that the antidepressant effect of ZZCD may be related to metabolic changes affecting protein kinase A (PKA)-cAMP-response element binding protein (CREB)—brain derived neurotrophic factor (BDNF)- tyrosine kinase receptor B (TrkB)—postsynaptic density protein 95 (PSD-95) pathway (Chai et al., 2021). However, detailed analysis of the antidepressant mechanism of ZZCD cannot be confirmed.

Recent evidence suggests that oxidative stress processes might play a relevant role in the pathogenesis of many major psychiatric disorders, including depression. Major depression has been associated with lowered concentrations of several endogenous antioxidant compounds, such as vitamin E, zinc and coenzyme Q10, or enzymes, such as glutathione peroxidase, and with an impairment of the total antioxidant status. Our recent research showed that the antidepressant of ZZCD was related to the glutathione (GSH) pathway. We found that ZZCD can reduce the toxicity of glutamate to PC12 cells, promote cell proliferation, inhibit apoptosis, scavenge oxygen free radicals, and enhance the activities of glutathione reductase (GR) and superoxide dismutase (SOD) antioxidant enzymes (Zhang et al., 2019).

GSH can directly react with reactive oxygen species (ROS) and nitrogen. Besides, act as a cofactor for GSH-S transferase and glutathione peroxidase, GSH is capable of exerting anti-oxidation and regulating nerves in the brain, promoting neurotransmitter transmission and neuron survival. The depletion of GSH is an indispensable part of the pathophysiology of depression (Morris et al., 2014). In addition, glutathione disulfide (GSSG) is the oxidized form of GSH, and the ratio of GSSG to GSH reflects the state of cellular redox balance (Haberhauer-Troyer et al., 2013), which is significantly increased in depressed patients (Papadopoulou et al., 2019).

The abnormal increase of ROS is related to a variety of diseases, including cancer, inflammation, and neurodegenerative diseases. The content of ROS in normal cells is strictly regulated to ensure redox homeostasis in the normal cells. Neuropsychiatric disorders, including depression, are also associated with telomerase shortening, nucleotide oxidation, and polymorphisms of several genes related to ROS. Mitochondrial dysfunction is directly related to the increase of oxidative stress. Oxidative stress plays an important role in the pathophysiological process of depression through the role of free radicals, non-free radical molecules, reactive oxygen species, and reactive nitrogen. Oxidative stress products represent significant parameters for measuring and predicting depression, and they are also important parameters for determining the effectiveness of taking antidepressants (Vaváková et al., 2015).

Altered brain structure plays an important role in the pathophysiology of depression, such as reduced volume and synapse number in the prefrontal cortex or hippocampus (Gerhard et al., 2016). In addition, dysfunction of this mood-related circuitry directly mediates numerous depressive symptoms (Duman et al., 2016). Therefore, it is likely that the measurement of the metabolic phenotype of the brain is a potential way to investigate the pathophysiology of depression. Spurred by a massive advance in analytical chemistry techniques combined with sophisticated statistical methods, metabolomics becomes a rapidly evolving field of life science (Rinschen et al., 2019). With the systematic, quantitative characterization of the complete collection of metabolites in a cell, organ, biofluid, or organism, metabolomics acts an important role in biomarker and mechanistic discoveries related to pathophysiological processes (Wishart, 2019). CUMS could induce representative and long-lasting symptoms of depressive behaviors, including anhedonia and despairing behavior in rodent animals (Wu et al., 2019). In addition, based on the favorable construct, face, and predictive validity, the CUMS model was reported to be widely used for researches on antidepressant efficacy and underlying mechanism (Akimoto et al., 2019). In this study, the brain metabolic spectrum was analyzed based on an ultra-performance liquid chromatography-quadrupole/time-of-flight mass spectrometry method. GSH, GSSG, ROS, SOD, GR, Glutathione peroxidase (GPx), and other metabolites related to oxidative stress in the rat brain were detected. In combination with previous cell metabolomics, the antidepressant mechanism of ZZCD in CUMS rats was elucidated from the aspects of untargeted metabolomics and targeted metabolomics.

## Materials and Methods

### Materials and Reagents

Dried ripe fruits of Gardenia jasminoides J.Ellis (Fructus Gardeniae) (No. 180525) and the fermented ripe seeds of Glycine max (L.) Merr. (Semen sojae praeparatum) (No. 180716-1) were purchased from Tong Han Chun Tang Chinese Herbal Factory (Shanghai, Chinese). Authenticated by Professor Lu-Ping Qin, Fructus Gardeniae (Voucher number 2018082001) and Semen sojae praeparatum (Voucher number 2018082002) were deposited at the herbarium of pharmaceutical analysis, School of Pharmacy, Second Military Medical University, Shanghai, China. Fluoxetine was purchased from Eli Lilly and Company (China). Sodium carboxymethyl cellulose (CMC-Na) and sucrose were obtained from Sangon Biotech Co., Ltd. (Shanghai). LC-MS grade formic acid, acetonitrile, and methanol were supplied by Honeywell (United States). Urethane was purchased from meilunbio (China). Deionized water was collected by a laboratory water purification system (HITECH Instruments CO., Ltd.). Reactive Oxygen Species, Glutathione and Glutathione disulfide, catalase, glutathione reductase, glutathione peroxidase, and SOD assay kit were purchased from Beyotime (China).

### Preparation of Zhi-Zi-Chi Decoction Extract

Gardeniae Fructus and Semen sojae praeparatum were powdered and sieved respectively. Then 600 g powder of ZZCD with a weight ratio of Gardeniae Fructus and Semen sojae praeparatum (2:1) was extracted twice by refluxing with 4,800 ml 50% ethanol for 1 h. Then the filtrate was mixed and centrifuged for 10 min at 3,000 r/min. After the supernatant was collected, the crude extract was prepared by evaporating to dryness by rotary vaporization at 60°C. Then the crude extract was dissolved and loaded on D101 microporous adsorption resins for 2 h. Different concentration of ethanol (0, 10, 20, 30, 40%) was used to wash resin. Then the 40% ethanol eluent was collected and concentrated by evaporation. The yield of ZZCD extract was about 5.02% (w/w), and the final yield of the extract partially purified was 2.1%.

### Qualitative and Quantitative Analysis

The powder of ZZCD used in this study is the same batch as our previous study. The qualitative analysis of ZZCD was performed on Agilent 6538 UHD Accurate-Mass Q-TOF LC/MS system. The quantitative analysis of ZZCD was performed on a Shimadzu HPLC system. Detailed information can be referred to our previous studies (Zhang et al., 2021).

### Method Validation of HPLC Analysis

Different methods were used to evaluate the specificity, linearity, precision, reproducibility, stability and recovery rate of the method. The specificity of the method was evaluated by analyzing blank solution (50% methanol), combining standard samples and ZZCD. The linearity was evaluated by using six different concentration analysis standard curves. The precision of the method was assessed by analyzing one pooled standard six times. The reproducibility of the method was assessed by analyzing six parallel pooled standards. One pooled standard was injected into the HPLC system at 0, 1, 2, 4, 8, 12, 24 h to determine the solution stability. The relative standard deviation of the peak area of the six compounds was calculated. An appropriate amount of ZZCD was divided into one part as a control group, and the other part was added with a labeled standard with an approximate concentration of ZZCD. After HPLC analysis, the recovery was calculated using the following equation: recovery (%) = (Total amount detected–original amount)/spiked amount × one hundred.

### Animal Treatments

All animal experiments were approved by the Ethics Committee of the Second Military Medical University (Shanghai, China). A total of 32 of male Sprague-Dawley rats (weighing 180–220 g) were obtained from Shanghai Sippr-BK laboratory animal Co. Ltd. (SCXK2013-0016). Under appropriate temperature (22 ± 2°C) and humidity (55 ± 5%), animals were adapted to the new experimental environment (12 h light-dark cycle) for 1 week. After that, the rats were divided into four groups with eight rats in each randomly: control (CON), model (CUMS), zhi-zi-chi decoction (ZZCD), and fluoxetine (Flu). The Flu group served as the positive group. For the next 7 weeks ( Zhao et al., 2020 ), all the rats were fed alone and followed the CUMS design except CON group (Supplementary Tables S1, S2). For the last 2 weeks, the rats in ZZCD group were given 5 g raw herb/kg ZZCD. In addition, the rats in Flu group were given 10 mg/kg fluoxetine, and the rats in CON and CUMS group were given CMC-Na solution. ZZCD and fluoxetine were dissolved in 0.5% CMC-Na solution. The bodyweight of all rats was recorded on day 1, 20, 36, and 52.

### Sucrose Preference Test

The sucrose preference test (SPT) was conducted on day 0, 22, 32, and 50. Before SPT, the rats were given two bottles of 1% sucrose solution for 24 h, then one of the bottles was replaced with water for another 24 h. In SPT, all rats were deprived of food and water for 23 h. After that, the rats were given a free choice of two bottles (one with 1% sucrose solution, the other with water) for 1 h. The weight of the consumed sucrose solution and water was measured to calculate the sucrose preference rate ( Willner et al., 1987; Deng et al., 2019). Sucrose preference rate (%) = consumed sucrose solution/(consumed sucrose solution + consumed water).

### Forced Swim Test

The forced swim test (FST) was conducted on day 0, 23, 33, and 51. The rats were separately placed in a cylinder (60 cm in height and 40 cm in diameter) filled with water at 25 ± 2°C to a depth of 40 cm. Every rat was adapted for 2 min and the total immobility time in the following 4 min was recorded. The immobility time was described as the amount of time that rats spent keeping their heads above the water without struggling ( Porsolt et al., 1977a; Jiang et al., 2017).

### Tail Suspension Test

The tail suspension test (TST) was conducted on day 0, 25, 32, and 52. With their heads 10 cm above the ground, the rats were suspended by their tails with adhesive tape from a ledge. The whole test lasted 6 min. Each rat was adapted for 2 min then the total immobility time in the remaining 4 min was recorded. The immobility time was defined as the amount of time that rats were suspended passively and remained completely motionless ( Steru et al., 1985; Wang et al., 2021).

### Sample Collection and Preparation

After the final TST, the rats were decapitated under anesthesia (10 mg/kg of 20% urethane). Because brain tissue is the direct target organ of stress stimulation, it can directly and completely reflect the changes of metabolites related to depression caused by a variety of stressors. Therefore, we chose the brain as the research object of analysis (Dang et al., 2022). The whole brain tissue was removed rapidly and quick-frozen by liquid nitrogen before stored at −80°C. We made sure that the entire set of operations was within 10 min. Prior to analysis, 100 mg brain tissue was thawed at room temperature and added with 1.5 ml cold 50% methanol. After homogenizing for 30 s at 5,000 rpm, the homogenate was centrifuged at 13,000 rpm/min for 10 min at 4°C. 800 μL supernatant was collected and then dried under nitrogen gas. The residues were redissolved in 100 μL 50% acetonitrile and vortexed for 3 min before centrifuged at 13,000 rpm/min for 10 min at 4°C. Then 50 μL supernatant was collected for analysis. The quality control (QC) sample was prepared by mixing 40 μL supernatant from each brain tissue sample.

### Metabolic Profiling Data Acquisition

Metabolic profiles of brain samples were obtained by Agilent 1,260/6,538 UHD Accurate-Mass Q-TOF LC/MS system (Agilent, United States). All samples were separated on XSelect HSS T3 column (100 mm × 2.1 mm, 2.5 μm) at 40°C. The mobile phase consisted of formic acid in water (0.1:100, v/v, A) and formic acid in acetonitrile (0.1:100, v/v, B) and operated under following program: 0–2 min, 5% B; 2–13 min, 5–95% B; 13–15 min, 95% B. The flow rate was set at 0.4 ml/min and the inject volume was 3 µL. The electrospray ionization source was used and the scan range was from 50 to 1,500 m/z in positive ion ionization mode. In addition, the nebulizer was set at 45 si and the flow rate of drying gas was set 11 L/min at 350°C.

### Method Validation

Following the same sample preparation and UHPLC-MS analysis, six QC samples were analyzed for six time to evaluate the precision and repeatability of the analytical method (Naz et al., 2014). The raw data of QC samples was imported to XCMS online for peak detection, retention time correction and alignment. Then 10 representative peaks covering different retention time and intensity were selected to calculate the relative standard deviation (RSD) of retention time and peak areas.

### Metabolomics Analysis

After UHPLC-MS raw data were imported to XCMS, a table containing retention time, peak areas, and m/z were obtained. Then the data were imported into Excel for peak area normalization. The metabolites in QC sample of which RSD of peak area below 20% and meet 80% rule were screened for multivariate statistical analysis. SIMCA (version 14.1, Umetrics, Sweden) was used to perform principal components analysis (PCA) and orthogonal partial least squares-discriminant analysis (OPLS-DA). The metabolites with the variable importance in the projection (VIP) beyond 1.0 and *p* value (t-test) below 0.05 were screened as differential metabolites. Then the metabolites were identified through HMDB, KEGG, Metlin, and related literature. At last, metabolic pathway analysis was conducted on MetaboAnalyst (version 4.0).

### Reactive Oxygen Species Determination

ROS level in brain samples was determined using ROS Assay Kit. DCFH-DA was a fluorescent probe, which could freely cross the cell membrane and be hydrolyzed by intracellular esterase to produce DCFH. ROS in brain sample could oxidize non-fluorescent DCFH to produce fluorescent DCF. And the detection of DCF fluorescence could be used to determine the level of ROS.

### Glutathione and Glutathione Disulfide Assay

GSH is a small peptide composed of three amino acid residues. GSSG is made by dehydrogenation of two GSHs. As a key antioxidant in animal cells, GSH is a major source of sulfhydryl groups in most living cells, which plays an important role in maintaining the proper redox state of sulfhydryl groups in proteins. After the homogenizing of brain samples, GSH and GSSG level in brain samples were determined by GSH and GSSG Assay Kit respectively.

### Measurement of the Activities of Reductase, GPx, and Superoxide Dismutase

As endogenous antioxidant defenses to deal with ROS, GR, GPx, and SOD were degrading enzymes to maintain the redox state of body. After the brain samples were homogenized for 30 s at 5,000 rpm, the homogenate was centrifuged at 13,000 rpm/min for 10 min at 4°C. Then the concentration of protein in supernatant was detected by bicinchoninic acid (BCA) protein assay kit. Then the activities of GR, GPx, and SOD were determined using GR, GPx, and SOD Assay Kit respectively.

### Statistical Analysis

Statistical analysis was conducted using SPSS (version 20.0, IBM SPSS Statistics, United States). All data were presented as mean ± standard deviation. The independent t-test was used to compare differences of peak areas of metabolites between two groups. The differences of results in behavior tests among four groups were analyzed by two-way repeated measures ANOVA. *p* < 0.05 was considered as statistically significant difference and *p* < 0.01 was considered as statistically extremely significant difference.

## Results

### Validation Results of HPLC Analysis

The results of method validation were shown in Supplementary Table S1. The results show that all the values are within the acceptance criteria, and the HPLC analysis method can be used to quantify the six compounds.

### Chemical Characterization Analysis

As shown in Supplementary Figure S1 and Supplementary Table S2, by comparison of mass spectrometry and fragment information with the literature, 25 species in ZZCD were identified. Among them, six key compounds were quantified by their calibration curves, including Genipin-1-β-D-gentiobioside, Geniposide, Daidzin, Glycitin, Genistin, and Daidzein (Supplementary Figure S2; Supplementary Table S3).

### Effect of Zhi-Zi-Chi Decoction on Body Wight and Ethology in Chronic Unpredictable Mild Stress Rats

**FIGURE 1:**
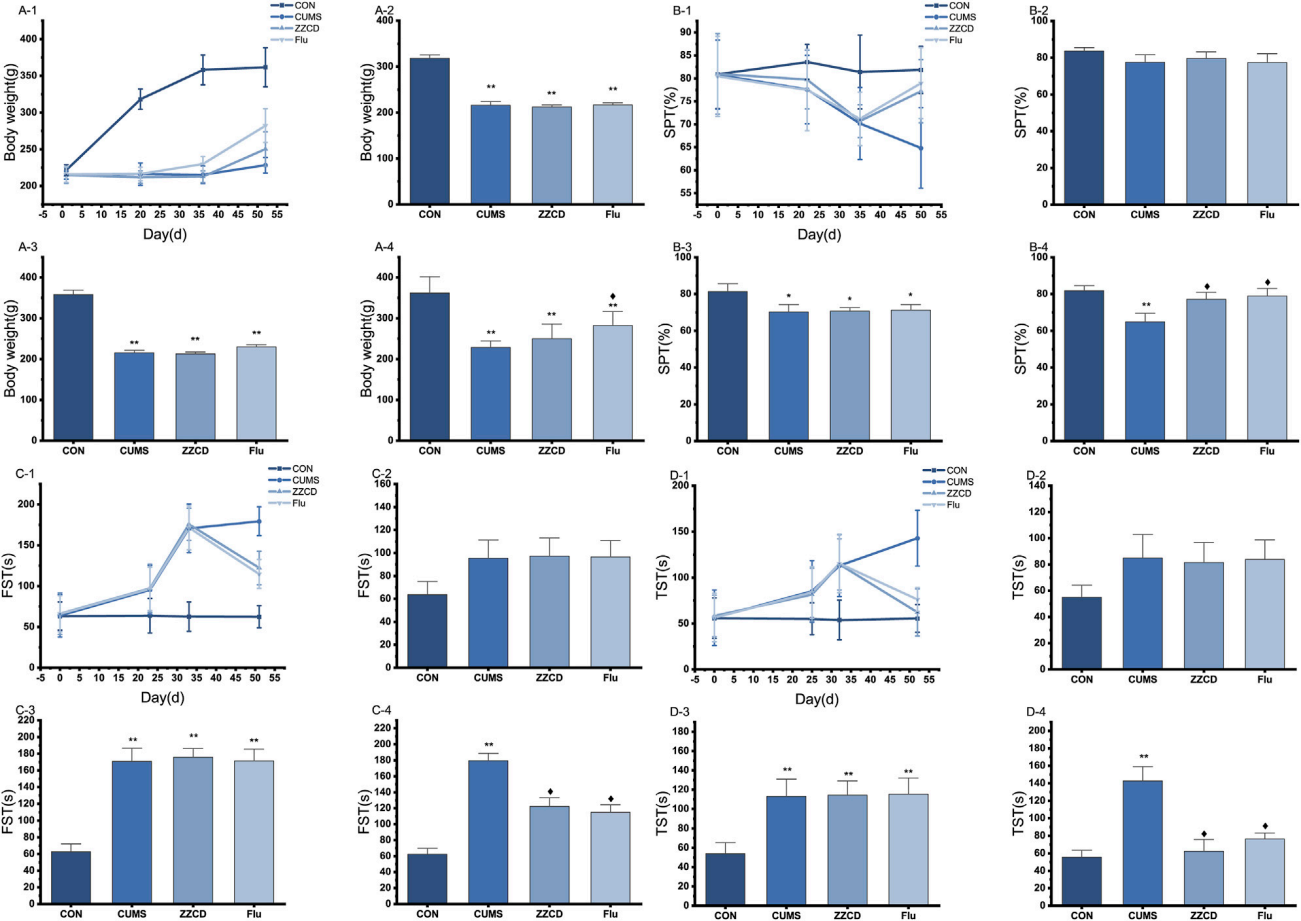
The change of body weight of rats in four group during 52 days **(A-1)**, on 20th day **(A-2)**, on 36th day **(A-3)**; on 52nd day **(A-4)**. The change of sucrose preference rate in four groups during 50 days **(B-1)**, on 22nd day **(B-2)**, on 35th day **(B-3)**; on 50th day **(B-4)**. (‾axis, *n* = 8). The change of immobility time in FST in four groups during 51 days **(C-1)**, on 23rd day **(C-2)**, on 33rd day **(C-3)**; on 51st day **(C-4)**. The change of immobility time in TST in four groups during 52 days **(D-1)**, on 25th day **(D-2)**, on 32nd day **(D-3)**; on 52nd day **(D-4)**. *p < 0.05 vs. CON; **p < 0.01 vs. CON; ♦*p* < 0.05 vs. CUMS.

The change of body weight of rats in four group was shown in Figure 1A. A statistical interaction was observed between different treatments and experiment duration (F = 43.462, *p* < 0.01). Compared with CON group, the body weight in CUMS group was decreased significantly from the 20th day (Mean Difference (MD) = −102.00, *p* < 0.01) and last to the 52nd day (MD = −133.38, *p* < 0.01). After treatment with fluoxetine for 2 weeks, the body weight was reversed significantly in Flu group on 52nd day (MD = 53.75, *p* < 0.05). However, the body weight in ZZCD group was not obviously increased on 52nd day (MD = 21.875, *p* = 0.074). As shown in Figure 1B, a statistical interaction between different treatments and experiment duration was observed (F = 2.157, *p* < 0.05). Compared with CON group, group CUMS sucrose preference rate from 35 days (MD = 11.21, *p* < 0.05) significantly reduced, and continued to day 50 (MD = 16.98, *p* < 0.01). After 2 weeks of treatment with ZZCD or fluoxetine, the sucrose preference was reversed in the ZZCD group (MD = 12.36, *p* < 0.05) and the Flu group (MD = 14.12, *p* < 0.05) on day 50. A statistical interaction was observed between different treatments and experiment duration (F = 11.23, *p* < 0.01) in Figure 1C. Compared with CON group, the immobility time in FST in CUMS group was significantly increased from the 33rd day (MD = 108.13, *p* < 0.01) and last to the 51st day (MD = 116.88, *p* < 0.01). After treatment with ZZCD or fluoxetine for 2 weeks, the immobility time in FST was reversed in ZZCD group (MD = −57.13, *p* < 0.05) and Flu group (MD = −64.50, *p* < 0.05) on the 51st day. As shown in Figure 1D, a statistical interaction between the different treatments and the duration of the experiment was observed (F = 5.489, *p* < 0.01). Compared with the CON group, CUMS group FST immobility time was significantly increased from Day 32 (MD = 59.13, *p* < 0.01), and continued until day 52 (MD = 87.38, *p* < 0.01). After treatment with ZZCD or fluoxetine for 2 weeks, was reversed in ZZCD group (MD = −80.75, *p* < 0.05) and Flu group (MD = −66.63, *p* < 0.05) on the 52nd day. After 2 weeks of treatment with ZZCD or fluoxetine, the immobility time in FST was reversed in the ZZCD group (MD = 12.36, *p* < 0.05) and the Flu group (MD = 14.12, *p* < 0.05) on day 50.

### Method Validation

In order to get a reproducible and sensitive result, method validation is essential. The results of precision and repeatability was shown in Table 1. The RSD of retention time and peak areas of the 10 representative peaks was below 1 and 10% respectively, indicating an acceptable and robust method to analyze the metabolic profiles of brain in rats was established.

**TABLE 1:** Stimulation method of CUMS model.

Validation parameter	RSD of retention time (%)	RSD of peak area (%)
Precision	0.86	9.62
Repeatability	0.8	9.35

### Zhi-Zi-Chi Decoction Reversed Metabolic Profiles of Brain in Chronic Unpredictable Mild Stress Rats

**FIGURE 2:**
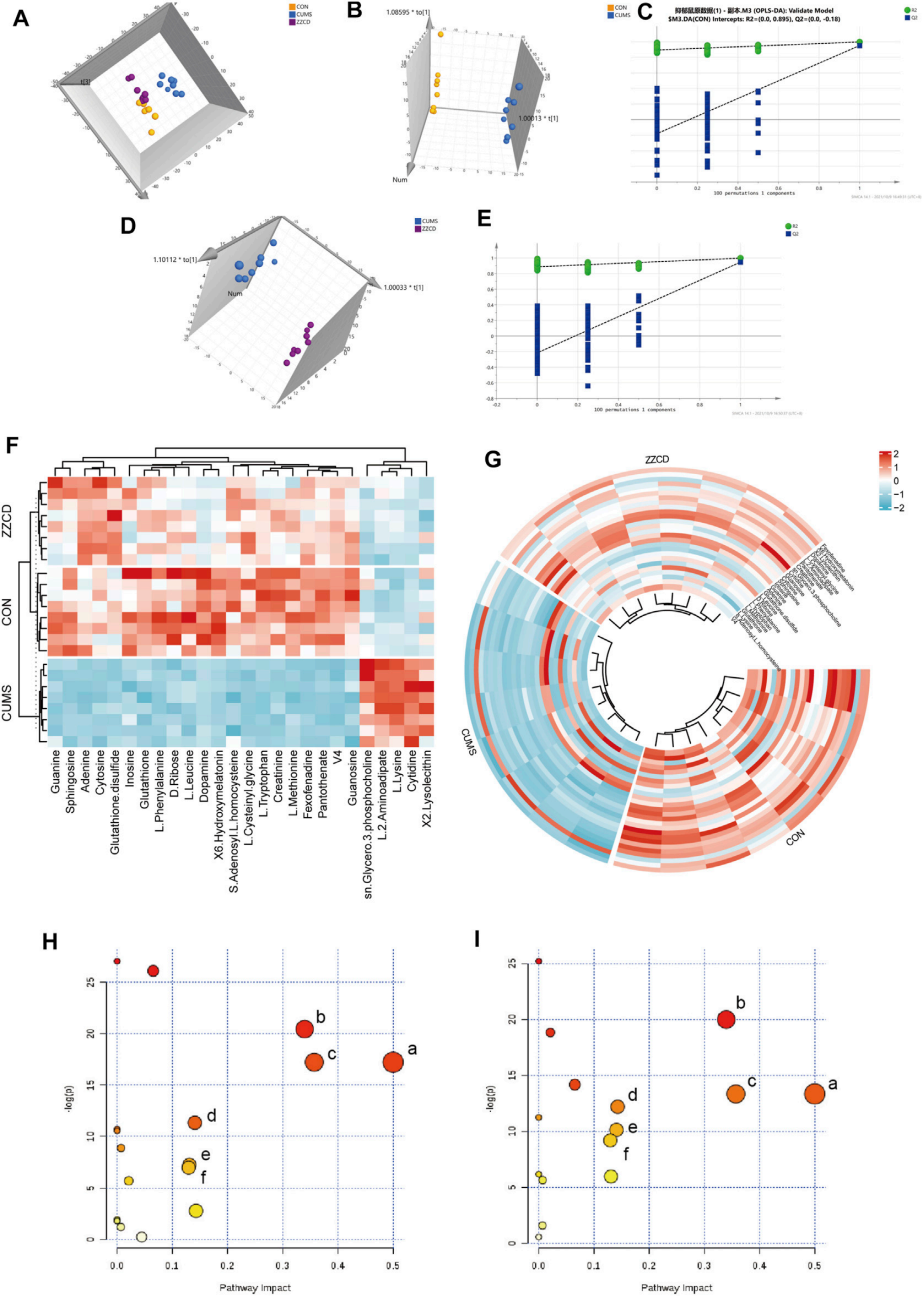
Multi-variate data analysis results. PCA scores plot of CON (yellow), CUMS (blue) and ZZCD (purple) group **(A)**, OPLS-DA scores plot of CON (yellow) and CUMS (blue) group **(B)**, Validation plot of CON and CUMS group by permutation tests **(C)**, OPLS-DA scores plot of CUMS (blue) and ZZCD (purple) group **(D)**, Validation plot of CUMS and ZZCD group by permutation tests **(E)**. Hierarchical clustering heat-map of the 26 differential metabolites **(F,G)**. Metabolic pathway analysis of CON vs. CUMS group, Phenylalanine, tyrosine and tryptophan biosynthesis (a), Glutathione metabolism (b), Phenylalanine metabolism (c), Lysine degradation (d), Cysteine and methionine metabolism (e), Tyrosine metabolism (f) **(H)** Metabolic pathway analysis of CUMS vs. ZZCD group, Phenylalanine, tyrosine and tryptophan biosynthesis (a), Glutathione metabolism (b), Phenylalanine metabolism (c), Tryptophan metabolism (d), Lysine degradation (e), Tyrosine metabolism (f) **(I)**.

To view the similarity and difference among three groups (Figure 2A), PCA was performed and obvious separation was observed between CON and CUMS group, CUMS and ZZCD group (R2X = 0.646, Q2 = 0.504). However, there was no obvious separation between CON and ZZCD group. Permutation test was used to evaluate the fit of the OPLS-DA model. As shown in Figures 2C,E, all simulations values on the left were smaller than the true value on the rightmost side, and Q2 had an intercept of less than 0.05 on the Y axis. This result indicated that the model was not overfitted. Then the significant differences were observed between CON and CUMS group (Figure 2B, R2X = 0.733, Q2 = 0.991), CUMS and ZZCD group (Figure 2D, R2X = 0.67, Q2 = 0.989). To discover the metabolites that play important role in classification among three group, both VIP value of OPLS-DA and *p* value of t-test were taken into consideration. After compared with HMDB, KEGG, Metlin, and related literature, 26 differential metabolites were screened (Table 2). Furthermore, obvious differences among three group could be seen intuitively at the hierarchical clustering heat-map (Figures 2F,G).

**TABLE 2:** Screened differential metabolites.

KEGG ID	Compound	RT (min)	m/z	VIP	*p* value	VIP	*p* value
				(CON vs. CUMS)	(CON vs. CUMS)	(CUMS vs. ZZCD)	(CUMS vs. ZZCD)
C00021	S-Adenosyl-L-homocysteine	0.97	385.1328	1.53	9.63E-10	1.56	3.01E-11
C00047	L-Lysine	0.57	147.1141	1.04	1.73E-05	1.06	2.38E-05
C00051	Glutathione	0.72	308.0959	1.33	1.91E-06	1.1	1.54E-05
C00073	L-Methionine	0.74	150.0598	1.28	6.97E-07	1.21	3.46E-06
C00078	L-Tryptophan	3.59	205.0984	1.34	2.99E-08	1.14	2.21E-05
C00079	L-Phenylalanine	1.88	166.088	1.91	2.32E-09	1.77	1.50E-08
C00121	D-Ribose	0.97	151.0644	1.6	1.64E-07	1.57	3.57E-08
C00123	L-Leucine	1.14	132.1035	1.06	6.95E-05	—	—
C00127	Glutathione disulfide	0.97	635.1502	1.3	9.28E-08	1.19	1.44E-06
C00147	Adenine	1.12	136.0632	1.24	2.53E-06	1.7	4.11E-07
C00242	Guanine	1.11	152.058	1.47	1.29E-06	1.87	3.33E-11
C00294	Inosine	0.74	269.0908	1.05	1.50E-05	1.01	3.30E-04
C00319	Sphingosine	13.18	322.2767	1.13	3.63E-05	—	—
C00380	Cytosine	0.71	112.0515	1.38	3.63E-08	1.25	1.73E-05
C00387	Guanosine	1.11	284.1021	1.48	2.44E-07	1.85	1.10E-08
C00475	Cytidine	0.96	244.0953	1.28	3.87E-06	1.27	5.21E-06
C00670	sn-Glycero-3-phosphocholine	0.7	258.1139	1.86	7.24E-10	2.02	9.29E-10
C00791	Creatinine	0.69	114.0673	—	—	1.26	2.11E-05
C00864	Pantothenate	2.44	220.1189	1.61	1.31E-07	1.45	2.37E-06
C00956	L-2-Aminoadipate	0.73	162.0776	1.89	2.36E-10	1.78	6.37E-12
C01419	L-Cysteinyl-glycine	0.96	179.0503	1.24	4.25E-06	1.41	3.28E-08
C03758	Dopamine	0.97	154.0877	1.58	1.49E-07	1.69	2.60E-07
C04230	2-Lysolecithin	10.44	496.3483	2.06	1.85E-08	1.27	1.32E-05
C05643	6-Hydroxymelatonin	1.12	271.1026	—	—	1.01	3.53E-04
C06999	Fexofenadine	10.14	502.3014	2.11	5.81E-10	1.73	9.87E-08
C13856	2-Arachidonoylglycerol	3.89	401.257	2.01	2.40E-09	2.03	2.92E-08

### Metabolic Pathway Analysis

**FIGURE 3:**
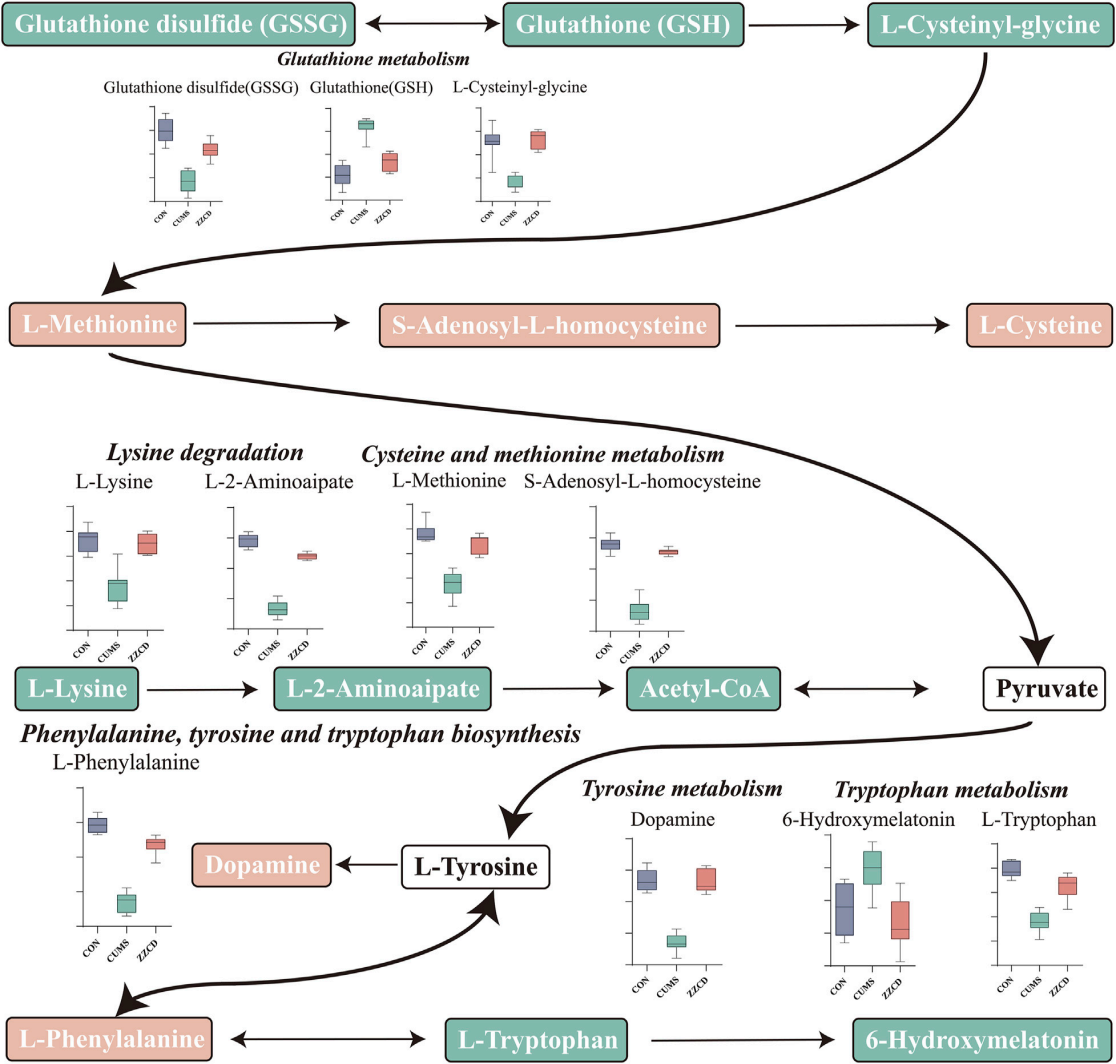
Schematic diagram of the altered differential metabolites and disturbed metabolic pathways.

MetaboAnalyst was a web-based tool for statistical, functional, and integrative analysis of metabolomics data. After pathway enrichment analysis and pathway topological analysis, the pathways with impact value >0.1, Holm adjust *p* < 0.01, and Raw *p* < 0.01 were screened as potential pathway contributed on antidepressive mechanism of ZZCD. There were seven metabolic pathways screened including Phenylalanine, tyrosine and tryptophan biosynthesis, Glutathione metabolism, Phenylalanine metabolism, Lysine degradation, Cysteine and methionine metabolism, Tyrosine metabolism, and Tryptophan metabolism (Tables 3, 4). To sum up, a schematic diagram of the changed metabolic pathways was shown in Figures 2H,I. The detailed pathway changes are presented in Figure 3.

**TABLE 3:** Summary of pathways with Metaboanalyst (CON vs. CUMS).

	Pathway name	Total compound	Hits	Raw p	Holm adjust p	Impact
A	Phenylalanine, tyrosine and tryptophan biosynthesis	4	1	3.30E-08	4.95E-07	0.5
B	Glutathione metabolism	28	3	1.32E-09	2.11E-08	0.33974
C	Phenylalanine metabolism	12	1	3.30E-08	4.95E-07	0.35714
D	Lysine degradation	25	2	1.20E-05	1.55E-04	0.14085
E	Cysteine and methionine metabolism	33	2	7.11E-04	6.40E-03	0.13105
F	Tyrosine metabolism	42	1	9.47E-04	7.58E-03	0.12972

**TABLE 4:** Summary of pathways with Metaboanalyst (CUMS vs. ZZCD).

	Pathway name	Total compound	Hits	Raw p	Holm adjust p	Impact
A	Phenylalanine, tyrosine and tryptophan biosynthesis	4	1	1.61E-06	1.77E-05	0.5
B	Glutathione metabolism	28	3	2.06E-09	2.88E-08	0.33974
C	Phenylalanine metabolism	12	1	1.61E-06	1.77E-05	0.35714
D	Tryptophan metabolism	41	2	5.05E-06	4.55E-05	0.14305
E	Lysine degradation	25	2	4.02E-05	2.81E-04	0.14085
F	Tyrosine metabolism	42	1	1.03E-04	6.17E-04	0.12972

### Effect of Zhi-Zi-Chi Decoction on Metabolites in Rat Brain

**FIGURE 4:**
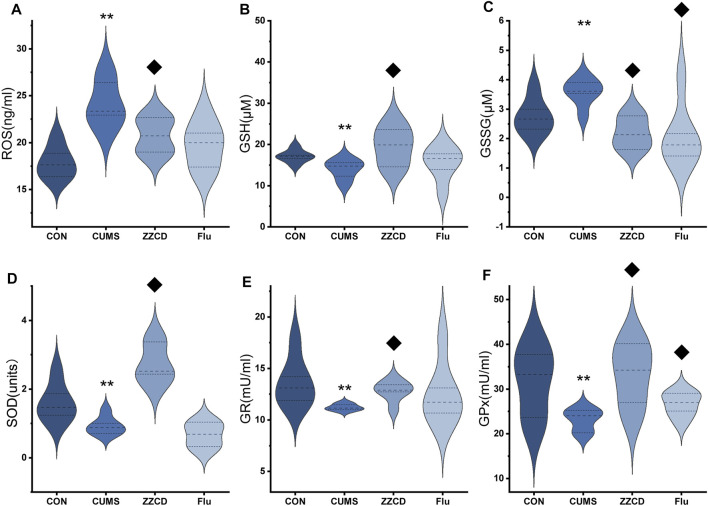
Effect of ZZCD on ROS **(A)** production, GSH **(B)** and GSSG **(C)** level, GR **(D)**, GPx **(E)**, and SOD **(F)** activities in brain samples of CUMS rats. ^**^ *p* < 0.01 vs. CON; ^♦^ *p* < 0.05 vs. CUMS.

In the brain of rats, the ROS level was analyzed. As the result shown in Figure 4A, compared to the CON group, the ROS level was significantly increased in CUMS group. Additionally, ZZCD decreased the ROS accumulation of the brain significantly as well as fluoxetine. As presented in Figures 4B,C, the results demonstrated that a significant decrease of GSH and an increase of GSSG happened to the rats exposed to CUMS in the brain. Treatment with ZZCD increased the declined level of GSH and decreased the elevated level of GSSG. In addition, fluoxetine could significantly reverse the change of GSSG level. To investigate the endogenous antioxidant defense of rats, the activities of catalase, GR, GPx, and SOD were measured. As provided in Figures 4D–F, CUMS stimulation caused the significantly reduced activities of catalase, GR, GPx, and SOD compared to the CON group. On the contrary, ZZCD could significantly up-regulate the activities of GR, GPx, and SOD.

## Discussion

So far, animal models of depression have played an important role in exploring the pathogenesis of depression. The CUMS rat simulated the clinical symptoms of most patients with depression by taking anhedonia as the key measurement index (Antoniuk et al., 2019), and current antidepressants could reverse its depressive behavior (Willner et al., 1987), which made it an ideal model for studying the pathogenesis of depression. In this study, CUMS stimulation combined with feeding alone was utilized to establish the animal model of depression. In addition, several tests including body weight, SPT, FST, and TST were conducted to evaluate the feasibility of the animal model of depression and the antidepressive effects of ZZCD on it.

According to the results of body weight, CUMS stimulation reduced the rate of weight gain in rats, which could be a characterization of depression (Yang et al., 2018). Anhedonia is the main internal phenotype in patients with depression. SPT could be used to assess the ability of the animal to experience pleasure, and a decrease in sucrose preference rate commonly indicated depression with anhedonia (Hales et al., 2014). When CUMS stimulation came to fifth week, the sucrose preference rate of rats in CUMS group was significantly decreased compared with those in CON group, reflecting the anhedonia status of the rats and indicating that the animal model of depression was successfully established. After 2 weeks of administration of ZZCD, the sucrose preference rate of rats in ZZCD group increased significantly, suggesting that ZZCD reversed the ability of rats to experience pleasure and exerted an antidepressive effect.

FST and TST were designed for the study of despair behavior in animal (Porsolt et al., 1977a; Steru et al., 1985), and the immobile state of rodents actually reflected an extinction-like inhibitory learning behavior caused by the inescapable feature of FST and TST (Campus et al., 2015). Drugs that reversed this immobility and stimulate escape behavior could be considered as therapeutic medications for depression (Porsolt et al., 1977b). When CUMS stimulation came to the fifth week, the immobility time of CUMS rats in FST and TST was significantly increased, which reflected the desperate state of the rats and indicated that the animal model of depression was successfully established. However, after 2 weeks of administration of ZZCD, the immobility time was significantly reduced, indicating that ZZCD stimulated escape the behavior of the rats, alleviated the state of despair, and exerted an antidepressant effect.

Taking the results of body weight, SPT, FST, and TST into consideration, the antidepressant effect of ZZCD on CUMS rats was discovered. In order to explore the underlying mechanism, the metabolic profiles in the brain of rats were analyzed using a verified metabolomics method based on UHPLC-Q/TOF-MS. Then the metabolic profiles in the brain among CON, CUMS, and ZZCD group were compared by multivariate statistical analysis, and 26 different metabolites associated with the antidepressant effect of ZZCD were screened. After metabolic pathway analysis, it was found that the antidepressant mechanism of ZZCD on CUMS rats related to the improvement of brain metabolism in Phenylalanine, tyrosine and tryptophan biosynthesis, Glutathione metabolism, Phenylalanine metabolism, Lysine degradation, Cysteine, and methionine metabolism, Tyrosine metabolism, and Tryptophan metabolism.

Glutathione (GSH) has crucial functions in the brain as an antioxidant, neuromodulator, neurotransmitter, and enable neuronal survival by reacting directly with reactive oxygen or nitrogen species, or by acting as an essential cofactor for GSH S-transferases and glutathione peroxidase. And GSH depletion plays an essential role in the pathophysiology of depression (Morris et al., 2014). In addition, glutathione disulfide (GSSG) is the oxidized form of GSH, and the ratio of GSSG to GSH reflects the state of cellular redox balance (Haberhauer-Troyer et al., 2013), which is significantly increased in patients with depression (Papadopoulou et al., 2019). Therefore, significantly decreased GSH and increased GSSG levels in the CUMS group revealed that CUMS stimulation may cause brain redox imbalance and neuronal injury in rats. Cys-Gly, a recognized ligand at the NMDA subclass of glutamate receptor, is negatively correlated with depression (Naumovski et al., 2010), which explains the reduction of Cys-Gly in CUMS group.

Betaine is a methylating agent in methionine circulation, which converts cysteine to methionine. The decrease of methionine in CUMS group indicated that CUMS may lead to the inhibition of methionine circulation, which was consistent with the increase of homocysteine levels in plasma and serum of patients with depression (Du et al., 2017). The decrease of lysine in CUMS group was consistent with the reported results of other studies on CUMS-induced rats (Liu et al., 2019). In addition, supplementation with lysine could significantly relieve anxiety (Zhao et al., 2015).

Phenylalanine is a neutral amino acid that can be converted to tyrosine by phenylalanine hydroxylase. And tyrosine can be further metabolized by tyrosine hydroxylase to a precursor of catecholamine neurotransmitter, which is closely related to depression. The significant reduction of phenylalanine in CUMS group could affect the synthesis of tyrosine and catecholamine neurotransmitters, resulting in the decrease of dopamine, and thus contributing to the pathogenesis of depression. In addition, tryptophan metabolism plays an important role in the course of depression. Low plasma content of tryptophan has been reported in patients with depression (Messaoud et al., 2019). and tryptophan could be used to treat depression (Messaoud et al., 2019). Therefore, the significant decrease of tryptophan in CUMS group indicated the pathological state of depression.

Compared with CUMS group, the content of brain metabolites in the ZZCD group was reversed to the normal level, and the disturbed metabolic pathways were restored, which suggesting the antidepressant effect of ZZCD on CUMS rats and revealing the underlying mechanism associated with the improvement of brain metabolism in Phenylalanine, tyrosine and tryptophan biosynthesis, Glutathione metabolism, Phenylalanine metabolism, Lysine degradation, Cysteine and methionine metabolism, Tyrosine metabolism, and Tryptophan metabolism.

PC12 cells have sympathetic phenotypes and are often used in neurobiological and neurobiochemical studies (Westerink and Ewing, 2008). The culture of PC12 cells does not need a special device, is easy to carry out, and eliminates the influence of blood, nerve, body fluid, and other factors. The growth environment is easy to be strictly controlled manually and has the advantages of both *in vitro* test and whole animal test. Chronic stress can lead to depressive nerve injury, and the release of excitatory amino acids is one of the pathological bases of stress anxiety, depression, and insomnia. Some studies have shown that PC12 cells stimulated by corticosterone, glutamate, and H_2_O_2_ can be used as a cell model for antidepressant research (Xie et al., 2013). Another previous study found that ZZCD can effectively reduce the toxicity of glutamate to PC12 cells, promote cell proliferation and inhibit glutamate-induced apoptosis. The results of ROS showed that ZZCD had the function of scavenging oxygen free radicals. In addition, ZZCD can also enhance the activities of GR and SOD antioxidant enzymes (Zhang et al., 2019). The impairment of glutamate-glutamine circulation in the hippocampus of patients with depression may lead to abnormal glutamate levels (Sanacora and Banasr, 2013). And the rapid antidepressant effect of antidepressants is related to its enhanced glutamate-glutamine cycle (Lee et al., 2013), which reveals the antidepressant effect of ZZCD based on neuroprotection. The imbalance of redox state is closely related to depressive symptoms (Sowa-Kućma et al., 2018), and antidepressants can correct the redox imbalance (Baek et al., 2016). In addition, the increase of oxidative stress and the impairment of antioxidant capacity can also reduce the neurogenesis and volume of the hippocampus (Filipović et al., 2017). By comparing the metabolomics results of the glutamate-induced PC12 cell damage model and the CUMS-induced depressed rat model, it was found that the two models had the same differential metabolites and common pathways, revealing a strong correlation between the glutamate-induced PC12 cell damage model and the CUMS-induced depressed rat model. Therefore, the reversal of the unbalanced redox state by ZZCD may also be the key to its antidepressant effect based on neuroprotection. In this study target metabolite changes of GSH/GSSG pathways metabonomics results verified the cells.

In the last decade, chronic stress has been considered as a vital risk factor for depression (Mcewen, 2017). And the role of oxidative damage caused by chronic stress has gained attention in depression. Under basal conditions, the brain consumes O2 and glucose by mitochondria to generate adenosine triphosphate (ATP), during which electrons bypass the formation of ATP and generate ROS (Herrero-Mendez et al., 2009). In situations of enhanced energy demand, the production of ATP is accompanied by an increase in mitochondrial ROS production (Murphy, 2009). Increased oxidative markers are observed in the brains of rodents subjected to different stress protocols such as chronic variable stress (Herbet et al., 2017). In our study, CUMS stimulation elevated ROS level in the brain, which could be associated with the increased energy demand caused by psychogenic stress. In addition, the activities of SOD, GR, and GPx in the brain was decreased significantly, which indicated a decrease in antioxidant defense and aggravated the ROS elevation. Except for degrading enzymes, some non-enzymatic compounds could also deal with ROS. Of these antioxidants, the most important and abundant is GSH, which protects against multiple oxidative insults (Mizui et al., 1992). However, the decreased GSH level following CUMS stimulation was observed in our study. Giving mitochondrial ROS overproduction and exhaustion of antioxidants, the brain was particularly susceptible to oxidative damage leading to structural integrity and cognitive function. Therefore, ZZCD reversing the ROS and antioxidants level indicated the antidepressive mechanism was closely related to antioxidant damage in the brain.

## Conclusion

In this study, brain metabolic profiling was investigated based on untargeted metabolomics to reveal the antidepressive mechanism of ZZCD on CUMS rats. The underlying antidepressant mechanism of ZZCD on CUMS rats was associated with the improvement of brain metabolism in Phenylalanine, tyrosine and tryptophan biosynthesis, Glutathione metabolism, Phenylalanine metabolism, Lysine degradation, Cysteine and methionine metabolism, Tyrosine metabolism, and Tryptophan metabolism. Combined with the results of cellular metabolomics, a targeted metabolomics analysis of the glutathione metabolic pathway was performed. The antidepressant effect of ZZCD is associated with its regulation of glutathione metabolism levels, reducing oxidative stress damage in the brain.

## Data Availability

The raw data supporting the conclusion of this article will be made available by the authors, without undue reservation.
