# Supplementary material for: Zhi-Zi-Chi Decoction Reverses Depressive Behaviors in CUMS Rats by Reducing Oxidative Stress Injury Via Regulating GSH/GSSG Pathway
Source: Front Pharmacol. 2022 Apr 7;13:887890. doi: 10.3389/fphar.2022.887890 (PMC9021728; doi:10.3389/fphar.2022.887890)
Supplement: Supplementary file 1 [file Table1.pdf]

Table S1. The results of method validation.

Compound	Regression equation	R^2	Precision(%)	Repeatability(%)	Stability(%)	Recovery(%)
Genipin-1-β-D-gentiobioside	y = 5,445.1x + 10,175	0.9999	1.7	2.0	1.3	0.5
Geniposide	y = 7,897.5x + 289,745	0.9995	1.2	0.3	0.8	1.0
Daidzin	y = 22,070x - 2,437.4	0.9999	1.3	0.3	0.5	1.5
Glycitin	y = 32,742x - 3,296.5	0.9999	1.1	1.8	3.6	1.9
Genistin	y = 36,003x - 7,412.6	0.9999	1.3	1.4	0.9	2.9
Daidzein	y = 35,387x - 1,998.9	0.9999	2.1	3.7	4.2	1.3
