# Supplementary material for: Zhi-Zi-Chi Decoction Reverses Depressive Behaviors in CUMS Rats by Reducing Oxidative Stress Injury Via Regulating GSH/GSSG Pathway
Source: Front Pharmacol. 2022 Apr 7;13:887890. doi: 10.3389/fphar.2022.887890 (PMC9021728; doi:10.3389/fphar.2022.887890)
Supplement: Supplementary file 2 [file Table2.pdf]

Table S2. MS date for characterization of compounds in ZZCD.

NO.	Formula	tR(min)	Molecular Weight	MS(+)	MS(-)	Compound Identification
1	$C_{16}H_{22}O_{11}$	3.366	390.1189		389.1114[M-H]-	Deacetylasperulosidic acid
2	$C_{16}H_{22}O_{10}$	3.962	374.1222	357.118[M+H-H2O]+	373.1154[M-H]-	Gardoside
3	$C_{16}H_{24}O_{11}$	3.962	392.133		373.1153[M-H-H2O]-	Shanzhiside
4	$C_{16}H_{18}O_{9}$	4.194	354.0961		353.0882[M-H]-	3-O-Caffeoylquinic acid
5	$C_{18}H_{26}O_{13}$	4.284	450.1389		449.1318[M-H]-	Gardenoside
6	$C_{23}H_{22}O_{11}$	4.284	474.1141		501.1014[M+HCOO-H2O]-	6"-O-Acetylgenistin
7	$C_{24}H_{22}O_{12}$	4.301	502.1096		501.1014[M-H]-	Malonyldaidzin
8	$C_{23}H_{34}O_{15}$	4.905	550.1909	573.1827[M+Na]+	549.1831[M-H]-	Genipin-1-β-D-gentiobioside
9	$C_{17}H_{24}O_{10}$	5.26	388.1344		433.1313[M+HCOO]-	Geniposide
10	$C_{17}H_{26}O_{11}$	5.26	406.145		433.1313[M+HCOO-H2O]-	Shanzhiside methylester
11	$C_{11}H_{14}O_{5}$	5.269	226.0843	227.0924[M+H]+	225.0766[M-H]-	Genipin
12	$C_{17}H_{26}O_{10}$	5.442	390.1508		435.1513[M+HCOO]-	Loganin
13	$C_{21}H_{20}O_{9}$	5.575	416.1117	417.1191[M+H]+	461.1103[M+HCOO]-	Daidzin
14	$C_{16}H_{26}O_{7}$	5.616	330.1686	331.1782[M+H]+	375.1665[M+HCOO]-	Jasminoside A
15	$C_{22}H_{22}O_{10}$	5.658	446.1199	447.1313[M+H]+	491.1189[M+HCOO]-	Glycitin
16	$C_{9}H_{8}O_{3}$	5.798	164.0477	147.0443[M+H-H2O]+	163.0403[M-H]-	P-coumaric acid
17	$C_{16}H_{26}O_{8}$	5.864	346.1624	329.1595[M+H-H2O]+	373.1499[M+HCOO-H2O]-	Jasminoside B
18	$C_{21}H_{20}O_{10}$	6.22	432.109	433.1163[M+H]+	477.1058[M+HCOO]-	Genistin
19	$C_{24}H_{22}O_{13}$	6.22	518.1041		545.0922[M+HCOO-H2O]-	Malonylgenistin
20	$C_{22}H_{36}O_{12}$	6.824	492.2216	510.2564[M+NH4]+	537.2215[M+HCOO]-	Jasminoside I
21	$C_{21}H_{34}O_{11}$	7.204	462.2116	485.203[M+Na]+	507.21[M+HCOO]-	Jasminoside T
22	$C_{15}H_{10}O_{4}$	7.353	254.0573	255.0668[M+H]+	253.0502[M-H]-	Daidzein
23	$C_{16}H_{12}O_{5}$	7.516	284.0698	285.0759[M+H]+		Glycitein
24	$C_{32}H_{44}O_{14}$	8.627	652.2735	675.2654[M+Na]+	697.2723[M+HCOO]-	beta-D-gentiobiosyl crocetin
25	$C_{10}H_{16}O_{3}$	9.09	184.1101	167.1072[M+H-H2O]+	165.0928[M-H-H2O]-	Jasminodiol
