# Supplementary figures and images for: Zhi-Zi-Chi Decoction Reverses Depressive Behaviors in CUMS Rats by Reducing Oxidative Stress Injury Via Regulating GSH/GSSG Pathway
Source: Front Pharmacol. 2022 Apr 7;13:887890. doi: 10.3389/fphar.2022.887890 (PMC9021728; doi:10.3389/fphar.2022.887890)

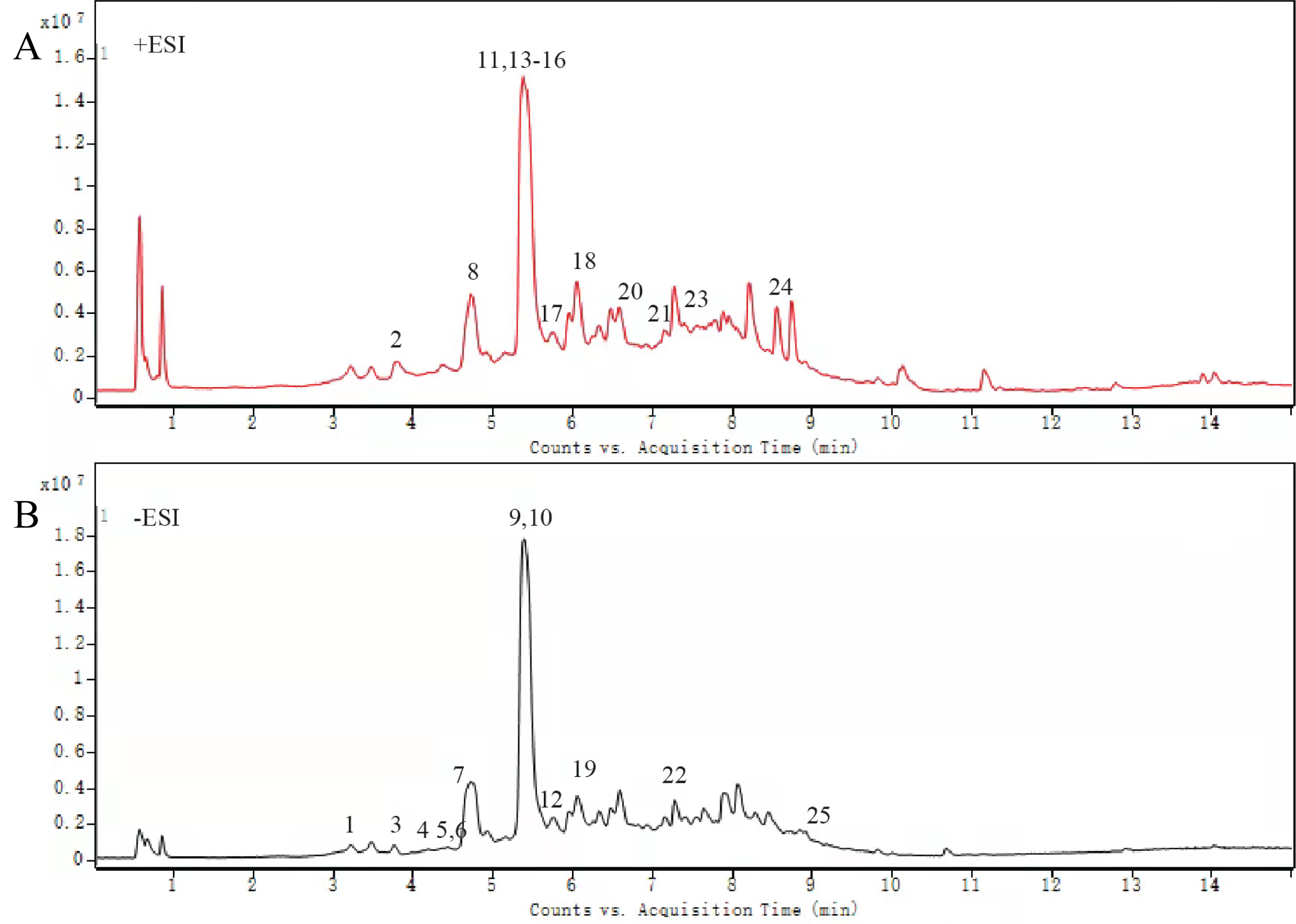

Supplement: Supplementary file 3 [file Image1.JPEG]

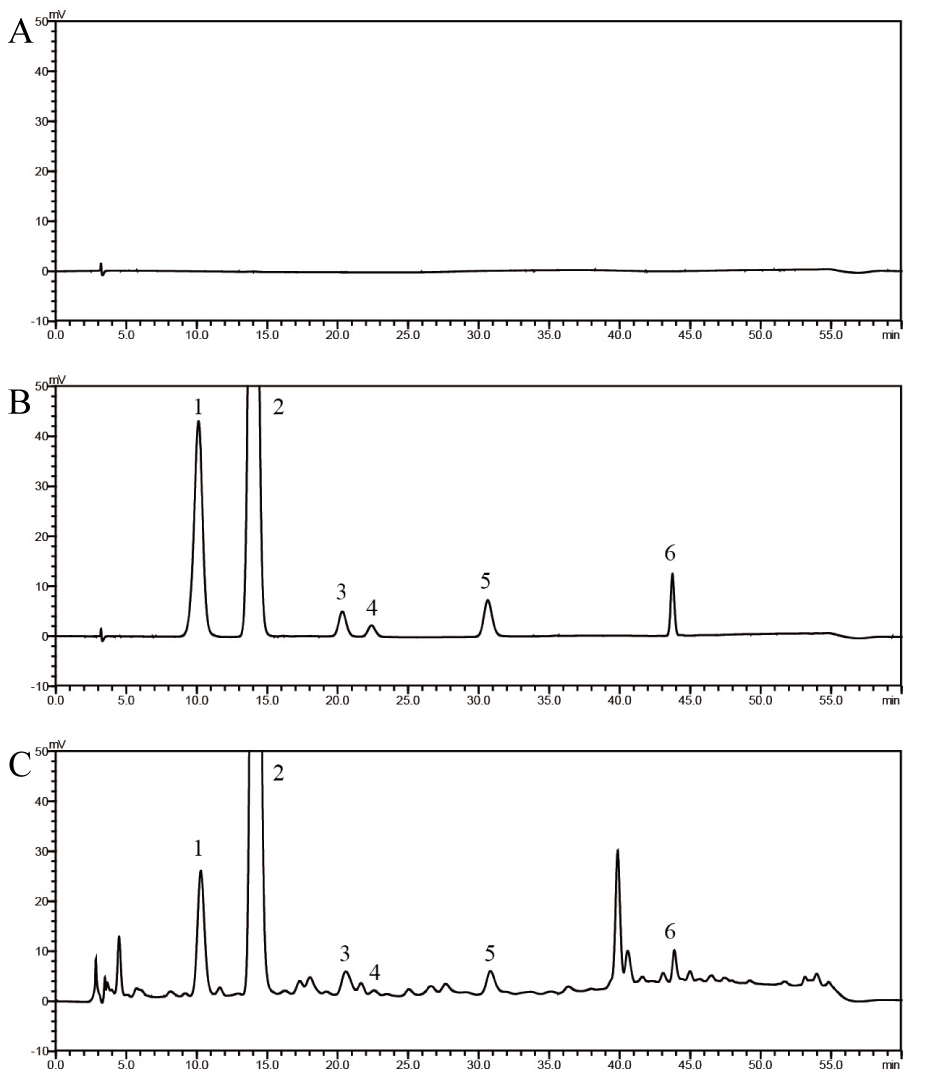

Supplement: Supplementary file 4 [file Image2.JPEG]
