# Supplementary material for: Zhi-Zi-Chi Decoction Reverses Depressive Behaviors in CUMS Rats by Reducing Oxidative Stress Injury Via Regulating GSH/GSSG Pathway
Source: Front Pharmacol. 2022 Apr 7;13:887890. doi: 10.3389/fphar.2022.887890 (PMC9021728; doi:10.3389/fphar.2022.887890)
Supplement: Supplementary file 5 [file Table3.pdf]

Table S3. The contents of six compounds in ZZCD.

NO	t_R (min)	Formula	Identified compound	Contents in ZZCD (mg/g)
1	10.12	$C_{23}H_{34}O_{15}$	Genipin-1-β-D-gentiobioside	36.010 ± 0.671
2	14.01	$C_{17}H_{24}O_{10}$	Geniposide	328.717 ± 0.540
3	20.33	$C_{21}H_{20}O_{9}$	Daidzin	2.062 ± 0.005
4	22.39	$C_{22}H_{22}O_{10}$	Glycitin	0.168 ± 0.003
5	30.64	$C_{21}H_{20}O_{10}$	Genistin	1.304 ± 0.020
6	43.74	$C_{15}H_{10}O_{4}$	Daidzein	1.160 ± 0.044
